# A cross-sectional study of evidence-based practice and association factors among nurses in public health facilities of Dessie city, Northeast Ethiopia

**DOI:** 10.3389/fpubh.2025.1540388

**Published:** 2025-03-11

**Authors:** Tariku Yimam, Asnakew Molla Mekonnen, Yawkal Tsega, Endalkachew Mesfin Gebeyehu

**Affiliations:** Department of Health System Management, School of Public Health, College of Medicine and Health Sciences, Wollo University, Dessie, Ethiopia

**Keywords:** evidence-based practice, nursing, public health facilities, Dessie city, Ethiopia

## Abstract

**Background:**

Evidence-based practice (EBP) is essential in modern healthcare to improve patient outcomes and enhance the quality of care. Nurses play a crucial role in implementing EBP in clinical settings, yet its utilization remains suboptimal in many healthcare facilities, particularly in resource-limited settings. However, nursing practice in Ethiopia often relies on experience, tradition, intuition, common sense, and untested theories. Additionally, there is a lack of information on the implementation of EBP by nurses in Ethiopia, particularly in the study area. However, there is limited empirical evidence assessing the level of EBP implementation and the associated factors among nurses in these facilities. Therefore, this study aims to assess evidence-based practice and association factors among nurses in public health facilities of Dessie city, Northeast Ethiopia.

**Methods:**

An institutional-based cross-sectional study was conducted among 442 nurses in public health facilities of Dessie City from January 17 to February 17, 2023. A stratified random sampling technique was used to select participants. Data were collected using a structured self-administered questionnaire and analyzed using bi-variable and multivariable binary logistic regression. Adjusted odds ratios (AOR) with 95% Confidence Intervals (CI) were calculated, and statistical significance was declared at *p* < 0.05.

**Results:**

The prevalence of good Evidence-Based Practice (EBP) utilization among nurses was 37.6% (95% CI: 32.9–42.2%), indicating a low level of adoption. The mean (±SD) age of the participants was 41.34 (±12.86) years. Key factors significantly associated with EBP implementation included: Knowledge of EBP: Nurses with good knowledge were six times more likely to implement EBP (AOR = 6.01; 95% CI: 3.78, 9.55). Type of Health Facility: Nurses working in hospitals were nearly three times more likely to practice EBP compared to those in health centers (AOR = 2.89; 95% CI: 1.45, 5.76). Attitude towards EBP: Nurses with a favorable attitude were 3.4 times more likely to engage in EBP (AOR = 3.41; 95% CI: 2.04, 5.71). Additionally, barriers to EBP adoption included limited resources (34.4%), high workload (27.5%), and lack of internet access at the workplace (68.7%). Less than 10% of nurses reported using nursing journals as sources of evidence, while 73.4% relied on information from coworkers.

**Conclusion:**

This study highlights a low prevalence of EBP utilization among nurses in Dessie City. The strong association between EBP adoption and factors such as knowledge, attitude, and type of health facility suggests the need for targeted interventions. Improving nurses’ access to EBP training, research resources, and institutional support could significantly enhance EBP implementation, leading to better patient care and health outcomes.

## Introduction

EBP is a cornerstone of modern healthcare, integrating clinical expertise, patient values, and the best available research evidence to enhance patient outcomes and overall healthcare quality (1). Nurses play a crucial frontline role in delivering care and ensuring patient safety, making their adoption of EBP particularly vital. However, despite its significance, EBP implementation remains inconsistent in many low-and middle-income countries, including Ethiopia, due to systemic, individual, and organizational challenges (2).

The EBP process involves several key steps, including formulating a clear clinical question using the Population/Patient Problem, Intervention, Comparison, and Outcome (PICO) format, searching for relevant literature, critically appraising the quality of evidence, integrating findings with clinical expertise and patient preferences, applying the evidence in practice, and evaluating outcomes to ensure effective healthcare interventions. However, several barriers hinder its widespread adoption among nurses, such as limited access to resources, inadequate training, and a lack of institutional support. Additionally, factors like nurses’ educational background, attitudes toward research, and workload significantly influence their ability to engage in evidence-based decision-making. Addressing these challenges through targeted interventions and enhanced institutional support is essential for fostering a culture of evidence-based nursing practice and improving patient care outcomes (1, 3).

Several studies have highlighted barriers to the adoption of EBP in nursing, including limited access to resources, inadequate training, and a lack of institutional support. These barriers significantly hinder nurses’ ability to implement EBP effectively (4). Additionally, factors such as nurses’ educational background, attitudes toward research, and workload play a crucial role in influencing their engagement in evidence-based decision-making (5, 6).

In Ethiopia, particularly in resource-limited settings like Dessie city, nursing practices frequently depend on traditional methods or outdated protocols rather than current evidence-based practices. This reliance on non-evidence-based approaches can lead to suboptimal patient care and outcomes. Public health facilities in these regions encounter several challenges that exacerbate the situation, including shortages of skilled personnel, heavy patient loads, and limited access to up-to-date medical literature (2, 7).

These barriers significantly hinder the implementation of EBP among nurses, making it crucial to assess the extent of EBP adoption and identify the factors that influence nursing practices in these settings. Understanding these factors is essential for developing targeted interventions that can promote EBP and ultimately enhance the quality of healthcare delivered to patients in Ethiopia (8).

EBP is a critical approach in modern healthcare, integrating clinical expertise, patient preferences, and the best available evidence to improve healthcare quality and patient outcomes. Nurses, as frontline healthcare providers, play a crucial role in implementing EBP to ensure safe, effective, and high-quality care. However, despite its proven benefits, the adoption of EBP among nurses remains inconsistent, particularly in low-resource settings like Ethiopia.

In Ethiopia, nursing practice is often influenced by experience, tradition, and intuition rather than current evidence-based guidelines. Many nurses rely on outdated protocols and personal judgment due to limited access to updated research findings, inadequate institutional support, and high patient workloads (9). Additionally, systemic challenges such as insufficient training on EBP, lack of mentorship, and restricted access to medical literature hinder nurses from fully integrating EBP into their clinical decision-making process (10, 11). These barriers contribute to variations in patient care quality, leading to suboptimal health outcomes (12).

Despite the growing emphasis on EBP in global healthcare, there is limited empirical evidence on its utilization among nurses in Ethiopia, particularly in Dessie City. Existing studies primarily focus on general healthcare quality, with few specifically addressing the extent of EBP adoption and the factors influencing its implementation among nurses in public health facilities (8). This knowledge gap underscores the need for a comprehensive assessment of EBP practice in this setting.

This study aims to assess the level of EBP adoption among nurses working in public health facilities in Dessie City, Northeast Ethiopia, and identify the key factors influencing its implementation. Specifically, the study seeks to:

- Examine the prevalence of EBP utilization among nurses in public health facilities.
- Identify the association between socio-demographic characteristics, institutional factors, and EBP adoption.

Despite increasing efforts to promote EBP in Ethiopia, there remains a critical knowledge gap regarding its adoption and the factors influencing its implementation in public health facilities. Understanding these factors is essential for developing targeted interventions, enhancing training programs, and improving institutional policies to support EBP adoption (8). Addressing these challenges can lead to better patient care and more effective nursing practices, ultimately improving health outcomes in Ethiopia.

The findings will provide evidence-based recommendations to policymakers and healthcare administrators to strengthen EBP integration into nursing practice in Ethiopia.

By systematically addressing these research gaps, this study will contribute to the growing body of knowledge on EBP adoption in resource-limited settings and inform future strategies to enhance evidence-based nursing care.

## Materials and methods

### Study setting, design, and period

This study employed an institutional-based cross-sectional design to assess the level of EBP utilization and its associated factors among nurses working in public health facilities in Dessie City, Northeast Ethiopia. Dessie City is located in the Amhara Regional State, serving as the administrative center of the South Wollo Zone. The study was conducted over a one-month period, from January 17 to February 17, 2023.

A total of 442 nurses were enrolled in this study, selected from two governmental hospitals (one general hospital and one comprehensive specialized hospital) and eight public health centers within Dessie City. The nurses were stratified based on their workplace, ensuring proportional representation from both hospitals and health centers. The majority of participants (357 nurses, 84%) were working in hospitals, while the remaining 68 nurses (16%) were employed in health centers. To ensure a representative sample, a stratified random sampling technique was used, where nurses were grouped based on their facility type, and participants were randomly selected from each stratum.

Data were collected using a structured self-administered questionnaire, covering socio-demographic information, knowledge and attitude towards EBP, institutional factors, and perceived barriers. The study aimed to capture a comprehensive understanding of the distribution of EBP adoption among nurses in different healthcare settings, considering variations in available resources, workload, and training opportunities. The findings from this study provide crucial insights into the challenges and facilitators of EBP implementation, ultimately guiding future policy decisions to improve evidence-based nursing practices in the region.

### Source and study population

All nurses working at public health facilities in Dessie city constituted the source population for the study. The study population included all sampled nurses who were selected during the study period at these facilities. Nurses who were on sick leave, annual leave, or maternity leave at the time of data collection were excluded from the study. Their absence from the workplace during the study period would limit their ability to provide accurate responses regarding their EBP practices and work environment. Nurses who were employed in administrative roles with no direct patient care responsibilities were not included in the study. Since the study aimed to assess the adoption of EBP in clinical settings, non-practicing nurses would not have direct exposure to patient care or decision-making processes related to EBP.

### Sample size estimation and sampling technique

The sample size was calculated using the single population proportion formula, taking a proportion of 55% for EBP based on a study conducted in referral hospitals in the Amhara region. This study indicated a confidence level of 95% and a margin of error of 5% (8, 11).


$$n=\frac{{\left(Za/2\right)}^{2}*p\left(1-p\right)}{d^{2}}$$

Where; *n* = the required sample size

P = proportion of EBP

a = level of confidence

z = degree of accuracy at 95%

d = margin of error


$$n=\frac{{\left(1.96\right)}^{2}*0.55\left(1-0.55\right)}{0.05^{2}}$$

n=380

The sample size for the second objective was determined using Epi Info software, version 7.2.3.1. This calculation considered the proportion (𝑝) in the unexposed group and the Adjusted Odds Ratio (AOR) as shown in Table 1.

**Table 1:** Sample size calculation procedure using Epi Info for evidence-based practice (EBP) and contributing factors among nurses working at public health facilities in Dessie city, Northeast Ethiopia, 2023.

Factors	Assumptions	Calculated sample size	Citation
Attitude towards EBP Yes	% outcome in unexposed group = 38.51%, AOR = 1.8, CI = 95%, Power = 80, Ratio = 1.26	402	(18)
Administrative support supportive	% outcome in unexposed group = 42.17%, AOR = 1.99, CI = 95%, Power = 80, Ratio = 2.9	381	(18)
Work experience 5–10 years	% outcome in unexposed group = 27.2%, AOR = 2.22, CI = 95%, Power = 80, Ratio = 0.56	264	(2)
Knowledge about EBP (Good)	% outcome in unexposed group = 37.54%, AOR = 2.04, CI = 95%, Power = 80, Ratio = 0.85	277	(19)

A total of 442 nurses were enrolled in this study, selected from two governmental hospitals (one general hospital and one comprehensive specialized hospital) and eight public health centers in Dessie City. Nurses were selected using a stratified random sampling technique to ensure proportional representation from hospitals and health centers. The sample size was determined using the single population proportion formula, with additional considerations for potential 10% non-response.

### Study variables and measurements

**Figure 1:**
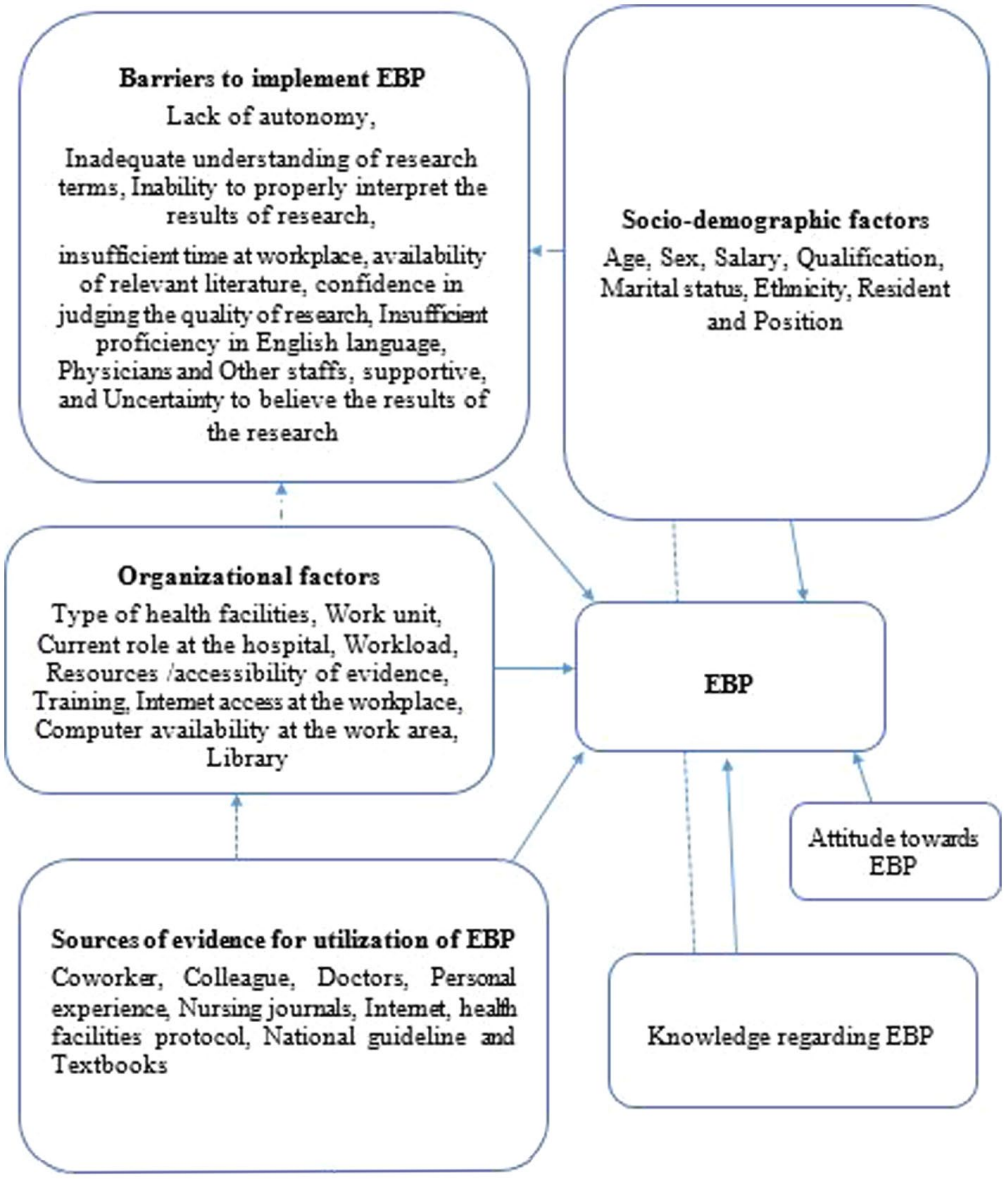
Conceptual frame work for EBP and its contributing factors among nurses working at public health facilities in Dessie city, Northeast Ethiopia 2023 adapted from different literature (9–12).

The outcome variable was EBP, categorized as either good or poor. Participants who scored at or above the mean cutoff point of 2.67 on the EBP questions—focused on the use of critically examined literature and research findings to provide safe and contemporary nursing care—were deemed to have good practice. In contrast, those who scored below this mean cutoff were classified as having poor practice (13, 14) (Figure 1).

#### Socio-demographic variables

The study assessed several socio-demographic variables, including age, sex, religion, ethnicity, marital status, work experience, educational level, and salary.

#### Barriers to implementing EBP

Participants reported various barriers to implementing EBP, such as a lack of autonomy, inadequate understanding of research terminology, difficulty interpreting research results, insufficient workplace time, limited access to relevant literature, lack of confidence in evaluating the quality of research, insufficient proficiency in English, inadequate support from physicians and other staff, and uncertainty in trusting research findings (3).

#### Knowledge

Participants who scored at or above the mean cutoff point of 1.82 on knowledge items related to EBP were classified as having good knowledge, while those who scored below this threshold were categorized as having poor knowledge (3, 14).

#### Sources for utilizing EBP

The sources for utilizing EBP included classrooms, hospital protocols, national guidelines, training sessions, colleagues, personal experience, doctors, nursing journals, the internet, and textbooks.

#### Organizational factors

The sources utilized for EBP included classrooms, hospital protocols, national guidelines, training sessions, colleagues, personal experience, doctors, nursing journals, the internet, and textbooks.

### Data collection instruments and procedures

Data were collected using a structured self-administered questionnaire adapted from various literature sources (11, 12, 15). The tool was initially prepared in English, then translated into Amharic (the local language). To ensure consistency, it was subsequently translated back into English.

The questionnaire comprised six sections with a total of 56 items. The first section gathered socio-demographic information using 11 items. The second section assessed nurses’ knowledge regarding EBP through 8 Yes or No questions. The third section focused on sources of information supporting EBP, containing 10 items rated on a six-point scale from “never” (1) to “always” (5). The fourth section included 7 items specifically related to EBP. The fifth section addressed barriers to utilizing EBP, consisting of 20 items constructed on a 5-point Likert scale ranging from “strongly disagree” (1) to “strongly agree” (5). Finally, the last section assessed attitudes toward EBP with 5 items.

## Data quality control

The questionnaire was initially developed in English, adopting variables and terminology from various studies. It was then translated into Amharic, the local language of the study area, and subsequently translated back into English by another expert to ensure consistency in meaning.

Before the main study, the questionnaire was pretested on 5% of the total sample size at the Kombolcha health facility, resulting in some modifications. The reliability of the checklist used to measure the outcome variable was assessed using the Cronbach’s alpha test, which yielded a reliability coefficient of 0.79.

Adequate orientation was provided to data collectors and study participants regarding the study’s objectives and the data collection procedures. The principal investigator checked the completed questionnaires on-site for consistency and completeness.

## Data processing and analysis

For data entry, EpiData version 4.6.0.2 (EpiData Association, Denmark) was used to ensure accurate and structured data management. After data entry, the dataset was exported to SPSS version 25 (IBM Corp., Armonk, NY, United States) for further statistical analysis. SPSS was used to perform descriptive statistics, bi-variable and multivariable logistic regression analyses to identify factors associated with EBP adoption among nurses. These software tools were selected for their reliability, efficiency, and widespread use in epidemiological and health research. Descriptive statistics, including frequency, percentage, median, and interquartile range, were computed and presented using frequency distribution tables, pie charts, and graphs.

To identify statistically significant associated factors, bi-variable logistic regression analysis was conducted for each independent variable in relation to the outcome variable. Variables with a *p*-value of less than 0.2 in the bi-variable analysis were included in the final model. Subsequently, multivariable logistic regression was performed, and variables with a *p*-value of ≤0.05 were considered significant factors. The results were presented using AOR along with 95% CI.

Model fit was assessed using the Hosmer-Lemeshow test, and multicollinearity was evaluated using a correlation matrix.

## Results

### Socio-demographic characteristics of participants

Out of the 442 nurses initially selected, 425 nurses participated in the study, yielding a response rate of 96.15%. The mean age of the participants was 41.34 years (±12.86). The majority of the nurses (61.4%) held a Bachelor of Science (BSc) degree, while 21% had a Master’s degree. There was no statistically significant association between socio-demographic variables such as age, educational qualification, or marital status and EBP utilization (*p* > 0.05) (Table 2).

**Table 2:** Socio-demographic characteristics of nurses working at public health facilities in Dessie city, Northeast Ethiopia, 2023 (*n* = 425).

Variables		Frequency	Percentage
Age group (years)	18–26 years old	87	20.5
	27-33 years old	114	26.8
	34–45 years old	140	32.9
	>45 years old	84	19.8
Qualification	C/Nurse	75	17.6
	BSc Nurse	261	61.4
	MSc	89	21
Ethnicity	Amhara	300	70.6
	Tigrie	38	8.9
	Oromo	52	12.2
	Afar	35	8.2
Marital status	Widowed	44	10.4
	Married	176	41.4
	Separated	34	8.0
	Divorced	43	10.1
	Single	128	30.1
Resident	Urban	316	74.4
	Rural	109	25.6
Salary in Ethiopian birr	<5,000	89	20.9
	5,000–10,000	188	44.2
	≥10,000	148	34.8

### Organizational factors

The majority (84%) of the respondents worked in hospitals, while 16% worked in health centers.

Nurses working in hospitals were nearly three times more likely to practice EBP than those in health centers (AOR = 2.89; 95% CI: 1.45, 5.76; *p* < 0.05), indicating a significant association.

34.4% of respondents reported insufficient resources as a barrier to EBP implementation, but this was not significantly associated with EBP utilization (*p* = 0.07).

61.6% of participants had received training on EBP, and among them, 40.8% practiced EBP. However, EBP training was not a statistically significant predictor of EBP utilization (AOR = 1.12; 95% CI: 0.60, 2.11; *p* > 0.05) (Table 3).

**Table 3:** Organizational factors affecting nurses working at public health facilities in Dessie city, Northeast Ethiopia, 2023 (*n* = 425).

Variables		Frequency (%)	EBP	
			Good (%)	Poor (%)
Working unit	Adult	218 (51.3%)	91 (21.4%)	127 (29.9%)
	Paediatrics	99 (23.3%)	32 (7.5%)	67 (15.8%)
	Emergency Unit	108 (25.4%)	37 (8.7%)	71 (16.7%)
Type of health facility	Hospital	357 (84.0%)	144 (33.9%)	213 (50.1%)
	Health centre	68 (16.0%)	16 (3.8%)	52 (12.2%)
Current role	Quality unit	41 (9.6%)	15 (3.5%)	26 (6.1%)
	Head nurse	71 (16.7%)	24 (5.6%)	47 (11.1%)
	Staff nurse	313 (73.6%)	121 (28.5%)	192 (45.2%)
Having workload	Yes	117 (27.5%)	38 (8.9%)	79 (18.6%)
	No	308 (72.5%)	122 (28.7%)	186 (43.8%)
Insufficient resources	Yes	146 (34.4%)	49 (11.5%)	97 (22.8%)
	No	279 (65.6%)	111 (26.1%)	168 (39.5%)
Working experience	<5 Years	50 (11.8%)	31 (7.3%)	19 (4.5%)
	> + 5 years	375 (88.2%)	234 (55.1%)	141 (33.2%)
Training on EBP	Yes	262 (61.6%)	107 (25.2%)	155 (36.5%)
	No	163 (38.4%)	53 (12.5%)	110 (25.9%)
Internet access at the workplace	Yes	133 (31.3%)	46 (10.8%)	87 (20.5%)
	No	292 (68.7%)	114 (26.8%)	178 (41.9%)
Computer availability at the work area	Available	106 (24.9%)	35 (8.2%)	71 (16.7%)
	Not available	319 (75.1%)	125 (29.4%)	194 (45.6%)
Library with updated references	Present	48 (11.3%)	16 (3.8%)	32 (7.5%)
	Absent	377 (88.7%)	144 (33.9%)	233 (54.8%)

### Sources of evidence for utilizing EBP

73.4% relied on information from coworkers, while only 6.8% used nursing journals.

The use of nursing journals was significantly associated with good EBP practice (*p* = 0.01) (Table 4).

**Table 4:** Sources of evidence for utilizing evidence-based practice among nurses working at public health facilities in Dessie city, Northeast Ethiopia, 2023 (*n* = 425).

Variables		Frequency (%)
Co-worker	Yes	312 (73.4)
	No	113 (26.6)
Health facilities protocols	Yes	86 (20.2)
	No	339 (79.8)
National guidelines	Yes	43 (10.1)
	No	382 (89.9)
Personal experience	Yes	236 (55.5)
	No	189 (44.5)
Doctors	Yes	120 (28.2)
	No	305 (71.8)
Nursing journals	Yes	29 (6.8)
	No	396 (93.2)
Internet	Yes	279 (65.6)
	No	146 (34.4)
Textbooks	Yes	118 (27.8)
	No	307 (72.2)

### Knowledge and attitude towards EBP

In the study, 41.9% of nurses demonstrated knowledge about evidence-based practice (EBP) and were six times more likely to implement it compared to those with poor knowledge (Adjusted Odds Ratio [AOR] = 6.01; 95% Confidence Interval [CI]: 3.78, 9.55; *p* < 0.001), indicating a strong positive association. Additionally, 64.9% of nurses held a favorable attitude towards EBP and were 3.4 times more likely to engage in it (AOR = 3.41; 95% CI: 2.04, 5.71; p < 0.001), reflecting a highly significant association.

**Figure 2:**
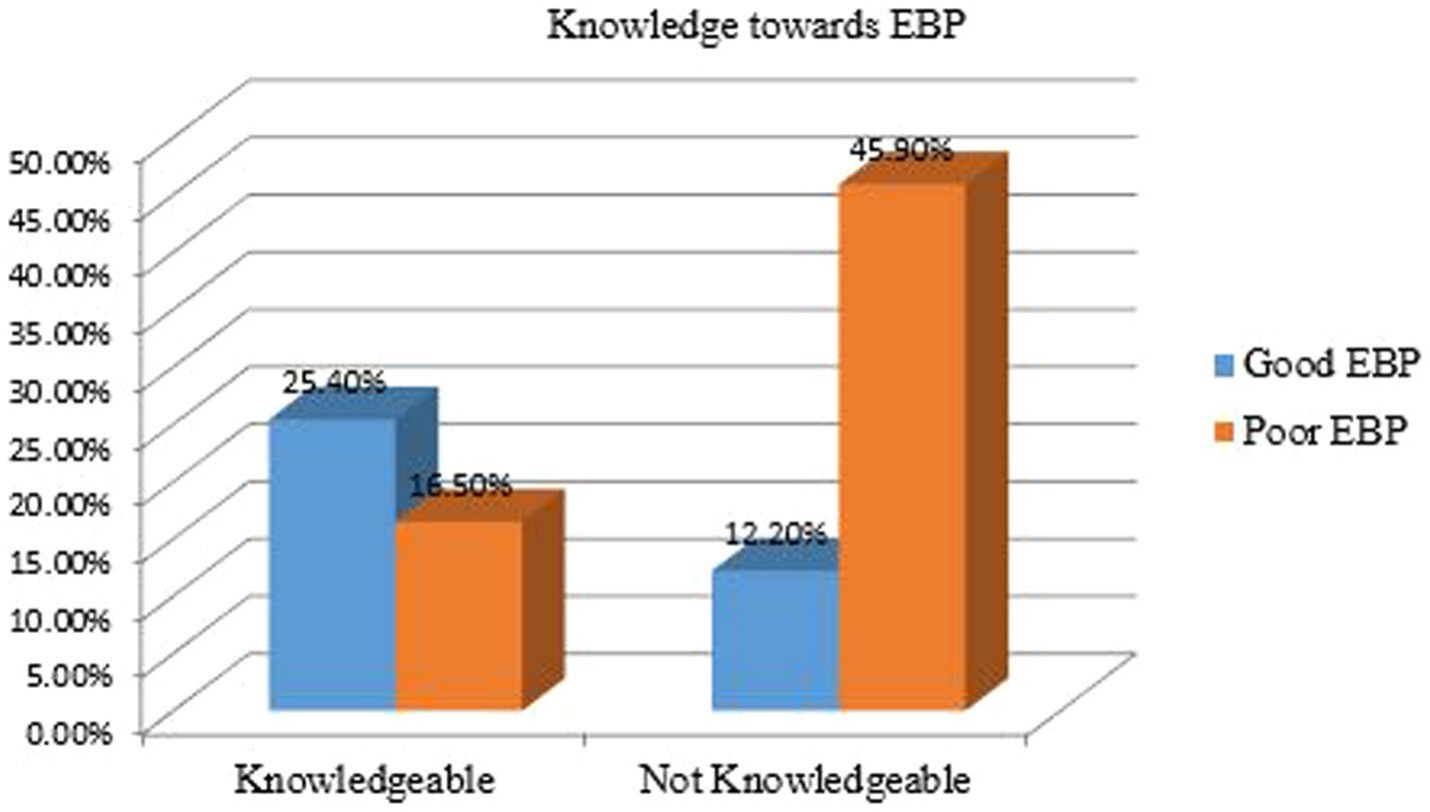
Knowledge of nurses regarding evidence-based practice at public health facilities Dessie city, Northeast Ethiopia, 2023 (*n* = 425).

**Figure 3:**
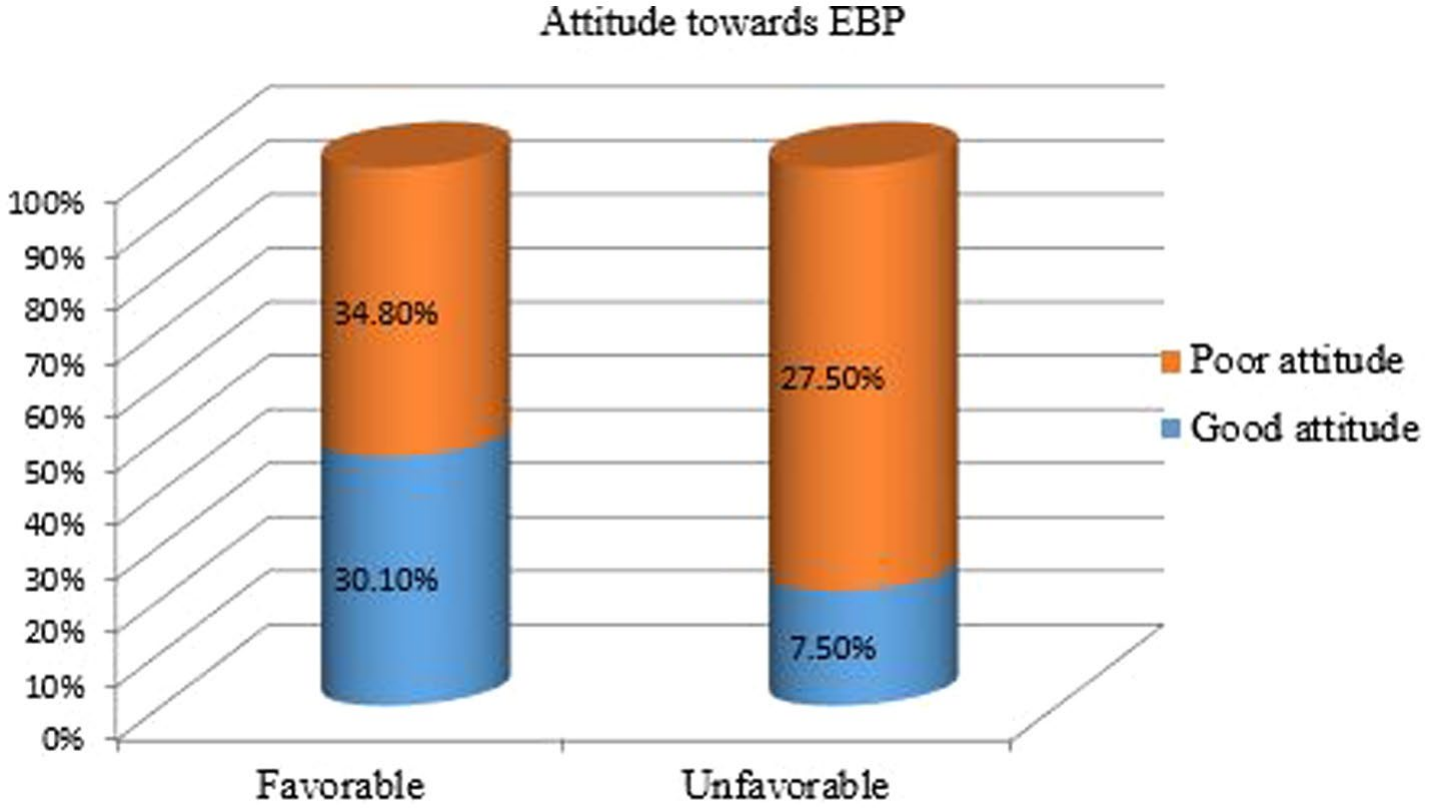
Attitude of the respondents towards EBP among nurses working at public health facilities in Dessie city, Northeast Ethiopia, 2023 (*n* = 425).

Figures 2, 3 illustrate the findings: Figure 2 presents the knowledge of nurses regarding EBP at public health facilities in Dessie city, Northeast Ethiopia, 2023 (*n* = 425), while Figure 3 depicts the attitudes of the respondents towards EBP among nurses working in the same facilities.

### Barriers to implementing EBP

Lack of cooperation from physicians (46.8%), insufficient English proficiency (44.7%), and limited access to literature (37.9%) were the most frequently reported barriers.

Perceived barriers were not significantly associated with EBP utilization (AOR = 0.87; 95% CI: 0.55, 1.38; *p* = 0.23) (Table 5).

**Table 5:** Barriers of the respondents to implement EBP among nurses working at public health facilities in Dessie city, Northeast Ethiopia, 2023 (*n* = 425).

Variables	Frequency (%)				
	1	2	3	4	5
Lack of autonomy to change practice	112 (26.4)	81 (19.1)	96 (22.6)	52 (12.2)	84 (19.8)
Inadequate understanding of research terms	31 (7.3)	16 (3.8)	33 (7.8)	143 (33.6)	202 (7.5)
Inability to properly interpret the results of research	34 (8.0)	26 (6.1)	53 (12.5)	152 (35.8)	160 (37.6)
Insufficient time at workplace to implement EBP	34 (8.0)	26 (6.1)	56 (13.2)	151 (35.5)	158 (37.2)
The relevant literature is not available	34 (8.0)	26 (6.1)	57 (13.4)	147 (34.6)	16 (37.9)
No confident in judging the quality of research	34 (8.0)	26 (6.1)	53 (12.5)	138 (32.5)	174 (40.9)
Insufficient proficiency in English language	36 (8.5)	19 (4.5)	39 (9.2)	141 (33.2)	190 (44.7)
Physicians are not cooperative with the implementation	35 (8.2)	16 (3.8)	29 (6.8)	146 (34.4)	199 (46.8)
Other staffs are not supportive to implement EBP	34 (8.0)	29 (6.8)	53 (12.5)	143 (33.6)	166 (39.1)
Uncertainty to believe the results of the research working to nurses’ practice	36 (8.5)	20 (4.7)	39 (9.2)	138 (32.5)	192 (45.2)
Perceived barrier (mean cut of point 3.85)	Yes	208 (48.9)			
	No	217 (51.1)			

1 = Strongly Disagree, 2 = Disagree, 3 = Neutral, 4 = Agree and 5 = strongly agree.

### Prevalence of EBP practice

**Figure 4:**
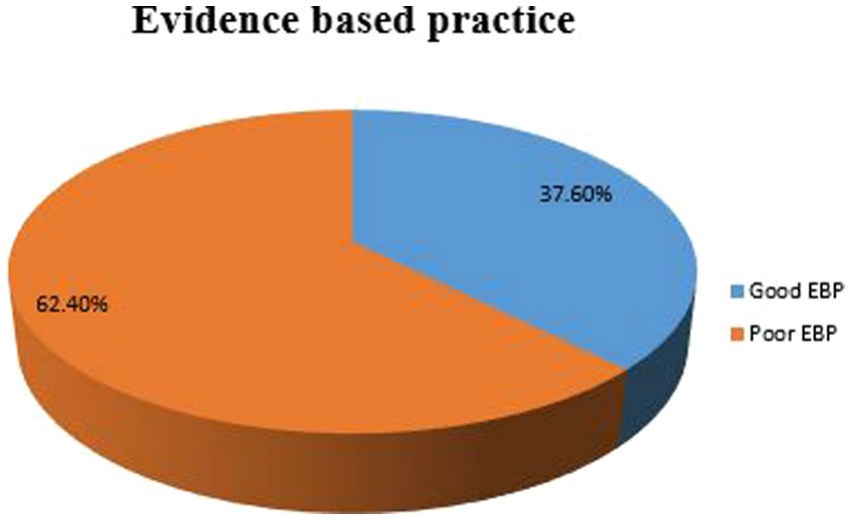
Evidence based practice among nurses working at public health facilities in Dessie city, Northeast Ethiopia, 2023 (*n* = 425).

The prevalence of good EBP practice among nurses in Dessie City was 37.6% (95% CI: 32.9–42.2%), indicating a low level of adoption. Evidence based practice among nurses shown in Figure 4: evidence based practice among nurses working at public health facilities in Dessie city, Northeast Ethiopia, 2023 (*n* = 425).

### Factors associated with EBP implementation

In the bi-variable analysis, several factors were found to be statistically significant at *p* < 0.2. These included qualification, age group, residency, respondents’ knowledge, type of health facility, training on evidence-based practice (EBP), attitude, workload, insufficient resources, and perceived barriers.

The multivariable logistic regression analysis revealed several key findings. Knowledgeable nurses were six times more likely to implement evidence-based practice (EBP) (AOR = 6.01; 95% CI: 3.78, 9.55; *p* < 0.001), indicating a strong positive association. Additionally, nurses working in hospitals were three times more likely to practice EBP compared to those in health centers (AOR = 2.89; 95% CI: 1.45, 5.76; *p* < 0.05), demonstrating a significant association. Furthermore, nurses with a favorable attitude toward EBP were 3.4 times more likely to engage in it (AOR = 3.41; 95% CI: 2.04, 5.71; *p* < 0.001), reinforcing the strength of this association (Table 6).

**Table 6:** Factors associated with EBP utilization among nurses in Dessie city, Ethiopia, 2023 (*n* = 425).

Variables		EBP		COR (95%CI)	AOR (95%CI)
		Good	Poor		
Qualification	C/Nurse	22	53	0.61 (0.32,1.17)	0.81 (0.37,1.78)
	BSc nurse	102	159	0.94 (0.57,1.54)	1.17 (0.66,2.06)
	MSc	36	53	1	1
Age group (years)	18–26 years old	30	57	1.05 (0.56,1.98)	1.00 (0.47,2.11)
	27-33 years old	43	71	1.21 (0.67,2.18)	1.29 (0.61,2.41)
	34–45 years old	59	81	1.45 (0.83,2.56)	1.53 (0.79,2.93)
	>45 years old	28	56	1	1
Resident	Urban	126	190	1.46 (0.92,2.32)	1.52 (0.86,2.67)
	Rural	34	75	1	1
Knowledge of the respondents	Knowledgeable	108	70	5.78 (3.76,8.88)	6.01 (3.78,9.55)**
	Not knowledgeable	52	195	1	1
Type of health facility	Hospital	144	213	2.19 (1.21,3.99)	2.89 (1.45,5.76)*
	Health center	16	52	1	1
Training on EBP	Yes	107	155	1.43 (0.95,2.16)	1.12 (0.60,2.11)
	No	53	110	1	1
Attitude	Favorable	128	148	3.16 (2.00,4.99)	3.41 (2.04,5.71)**
	Unfavorable	32	117	1	1
Having workload at workplace	Yes	38	79	0.73 (0.46,1.15)	0.64 (0.38,1.07)
	No	122	186	1	1
Insufficient resources at workplace	Yes	49	97	0.76 (0.50,1.16)	0.70 (0.43,1.14)
	No	111	168	1	1
Perceived barrier	Yes	76	131	0.92 (0.62,1.37)	0.87 (0.55,1.38)
	No	84	134	1	1

NB: * indicate at *p*-value < 0.05, ** indicate at *p*-value < 0.001 and 1 indicate reference category.

## Discussion

This study identified a low prevalence of Evidence-Based Practice (EBP) utilization among nurses in Dessie City, Ethiopia, with only 37.6% demonstrating good EBP practices. Key factors significantly associated with EBP implementation included nurses’ knowledge, the type of health facility, and their attitudes toward EBP. Specifically, nurses with good knowledge were six times more likely to implement EBP effectively, while those working in hospitals were nearly three times more likely to engage in EBP compared to their counterparts in health centers. Interpreting these findings suggests that knowledge is a crucial determinant of EBP adoption. The strong association between knowledge and EBP utilization highlights the importance of education and training in enabling nurses to incorporate research findings into their clinical practice. This aligns with the principle that well-informed healthcare professionals can significantly enhance patient outcomes (16).

When compared to other studies, this research mirrors findings from similar contexts. For instance, a study conducted in Kenya reported an EBP prevalence of 12% among nurses, indicating challenges in implementing EBP across low-resource settings (11). In Ghana, a slightly higher EBP utilization rate of 25.3% was observed, suggesting varying degrees of adoption influenced by local circumstances (12). These comparisons illustrate a common struggle faced by nurses in resource-limited environments regarding EBP adoption.

The differences in EBP adoption rates across studies may stem from several factors. Access to training, resources, and institutional support can vary widely between countries and regions. For example, nurses in higher-performing settings may benefit from better access to educational resources and mentorship, which can facilitate EBP adoption. Furthermore, cultural attitudes toward research and evidence can also impact nurses’ willingness to engage in EBP (17). In Ethiopia, systemic challenges such as high workloads and insufficient institutional support contribute to the low EBP utilization observed in this study.

The implications of these findings are significant. The low prevalence of EBP among nurses in Dessie City underscores the urgent need for targeted interventions aimed at enhancing EBP education and resources. Improving access to training programs, fostering a supportive work environment, and promoting a culture of evidence-based nursing are essential steps toward increasing EBP adoption. Policymakers and healthcare administrators should prioritize the development of structured training initiatives and resource allocation to create an environment conducive to EBP (4).

### Strengths and limitations of the study

The study achieved a 96.15% response rate, ensuring high data completeness and reliability. A stratified random sampling technique was employed, ensuring a representative sample of nurses from both hospitals and health centers in Dessie City. The questionnaire was adapted from previously validated studies and pretested to ensure clarity, reliability, and consistency.

The study did not include qualitative methods, such as in-depth interviews or focus group discussions, which could have provided deeper insights into barriers and facilitators of EBP. Some responses related to EBP practice and training history may have been influenced by recall bias, affecting data accuracy.

## Conclusion

This study highlights a concerningly low prevalence of EBP utilization among nurses in Dessie City, Ethiopia, with only 37.6% demonstrating effective EBP practices. The findings reveal that knowledge, type of health facility, and attitudes toward EBP are significant factors influencing its implementation. Specifically, nurses with good knowledge were six times more likely to engage in EBP, and those working in hospitals were nearly three times more likely to practice EBP compared to those in health centers.

These results underscore the urgent need for targeted interventions to enhance EBP education and resources within the nursing workforce. Addressing systemic barriers, such as limited access to training and institutional support, is essential for fostering a culture of evidence-based nursing practice. By implementing comprehensive training programs and promoting supportive work environments, healthcare administrators can significantly improve EBP adoption, leading to better patient care and health outcomes in the region.

## Data Availability

The original contributions presented in the study are included in the article/supplementary material, further inquiries can be directed to the corresponding author.
